# High resolution melting curve analysis targeting the HBB gene mutational hot-spot offers a reliable screening approach for all common as well as most of the rare beta-globin gene mutations in Bangladesh

**DOI:** 10.1186/s12863-017-0594-3

**Published:** 2018-01-02

**Authors:** Md Tarikul Islam, Suprovath Kumar Sarkar, Nusrat Sultana, Mst. Noorjahan Begum, Golam Sarower Bhuyan, Shezote Talukder, A. K. M. Muraduzzaman, Md Alauddin, Mohammad Sazzadul Islam, Pritha Promita Biswas, Aparna Biswas, Syeda Kashfi Qadri, Tahmina Shirin, Bilquis Banu, Salma Sadya, Manzoor Hussain, Golam Sarwardi, Waqar Ahmed Khan, Mohammad Abdul Mannan, Hossain Uddin Shekhar, Emran Kabir Chowdhury, Abu Ashfaqur Sajib, Sharif Akhteruzzaman, Syed Saleheen Qadri, Firdausi Qadri, Kaiissar Mannoor

**Affiliations:** 1 Laboratory of Genetics and Genomics, Institute for Developing Science and Health Initiatives, Mohakhali, Dhaka, Bangladesh; 2 Infectious Diseases Laboratory, Institute for Developing Science and Health Initiatives, Mohakhali, Dhaka, Bangladesh; 3 Department of Virology, Institute of Epidemiology, Disease Control and Research, Mohakhali, Dhaka, Bangladesh; 4 Department of Paediatric Medicine, KK Women’s and Children’s Hospital, 100 Bukit Timah Road, Singapore, Singapore; 5 Department of Biochemistry and Molecular Biology, Dhaka Shishu Hospital, Dhaka, Bangladesh; 6 Department of Neonatology, Bangabandhu Sheikh Mujib Medical University, Shahbag, Dhaka, Bangladesh; 7 Department of Biochemistry and Molecular Biology, University of Dhaka, Dhaka, Bangladesh; 8 Department of Genetic Engineering & Biotechnology, University of Dhaka, Dhaka, Bangladesh; 9 Department of Enteric and Respiratory Infectious Diseases, Infectious Diseases Division, International Centre for Diarrhoeal Disease Research, Bangladesh, Mohakhali, Dhaka, Bangladesh

**Keywords:** Beta-globin gene, Mutational hot-spot, Beta-thalassemia, High resolution melting curve, Carrier screening

## Abstract

**Background:**

Bangladesh lies in the global thalassemia belt, which has a defined mutational hot-spot in the beta-globin gene. The high carrier frequencies of beta-thalassemia trait and hemoglobin E-trait in Bangladesh necessitate a reliable DNA-based carrier screening approach that could supplement the use of hematological and electrophoretic indices to overcome the barriers of carrier screening. With this view in mind, the study aimed to establish a high resolution melting (HRM) curve-based rapid and reliable mutation screening method targeting the mutational hot-spot of South Asian and Southeast Asian countries that encompasses exon-1 (c.1 - c.92), intron-1 (c.92 + 1 - c.92 + 130) and a portion of exon-2 (c.93 - c.217) of the HBB gene which harbors more than 95% of mutant alleles responsible for beta-thalassemia in Bangladesh.

**Results:**

Our HRM approach could successfully differentiate ten beta-globin gene mutations, namely c.79G > A, c.92 + 5G > C, c.126_129delCTTT, c.27_28insG, c.46delT, c.47G > A, c.92G > C, c.92 + 130G > C, c.126delC and c.135delC in heterozygous states from the wild type alleles, implying the significance of the approach for carrier screening as the first three of these mutations account for ~85% of total mutant alleles in Bangladesh. Moreover, different combinations of compound heterozygous mutations were found to generate melt curves that were distinct from the wild type alleles and from one another. Based on the findings, sixteen reference samples were run in parallel to 41 unknown specimens to perform direct genotyping of the beta-thalassemia specimens using HRM. The HRM-based genotyping of the unknown specimens showed 100% consistency with the sequencing result.

**Conclusions:**

Targeting the mutational hot-spot, the HRM approach could be successfully applied for screening of beta-thalassemia carriers in Bangladesh as well as in other countries of South Asia and Southeast Asia. The approach could be a useful supplement of hematological and electrophortic indices in order to avoid false positive and false negative results.

**Electronic supplementary material:**

The online version of this article (10.1186/s12863-017-0594-3) contains supplementary material, which is available to authorized users.

## Background

The hemoglobinopathies are a group of inherited monogenic disorders of erythrocytes that affect millions of people worldwide [1, 2]. These disorders are caused by mutation(s) of α or β globin gene of hemoglobin that result in a missing or a defective production of the oxygen-transport protein [3]. Beta thalassemia is characterized by an abnormal or absent production of hemoglobin due to mutation(s) in the protein-coding part or the intron of the beta-globin gene or its promoter in chromosome 11 [4–6]. There are at least 893 beta-globin gene mutations reported till date (http://globin.bx.psu.edu/cgi-bin/hbvar/query_vars3). Even though large numbers of beta-globin gene mutations have been reported throughout the world, the β-thalassemia patients from each ethnic population carry only a limited number of mutations which comprise of some frequently occurring mutations as well as some rare mutations. For example, five common mutations, namely c.92 + 5G > C, c.92 + 1G > T, c.126_129delCTTT, c.27_28insG and the 619 bp deletion have been reported to account for over 90% of the mutations in beta-thalassemia patients in India [7, 8]. Similar to India, only five mutations, including c.-78A > G, c.52A > T, c.126_129delCTTT, c.216_217insA and c.316-197C > T in HBB gene are responsible for 90% of beta-thalassemia patients in china [9].

The majority of the beta-thalassemia mutations in Bangladeshi population have been reported to originate in the neighboring India, Southeast Asian countries, Sri Lanka, Pakistan etc. [10–13]. In Bangladesh, the most frequently occurring mutations, namely c.79G > A, c.92 + 5G > C and c.126_129delCTTT; and the less common mutations c.92G > C, c.27_28insG, c.47G > A, c.92G > A, c.46delT, c.92 + 130G > C, and c.51delC have been accounted for more than 95% of beta thalassemia patients [13, 14]. All of these mutations together constitute the mutational hot-spot (c.1 – c.92 of exon 1, c.92 + 1 – c.92 + 130 intron-1 and c.93 – c.217 of exon-2) in the HBB gene of Bangladeshi thalassemia patients. The same hot spot harbors majority of HBB gene mutations in many other countries of South Asia and Southeast Asia. Hence, the hot-spot could be a useful target to screen the beta-globin gene mutations and therefore can serve as a supplement to hematological and electrophoretic indices to overcome certain limitations of these approaches. For instance, the screening of beta-thalassemia carrier which is based solely on hematological or electrophoretic indices may miss a significant number of carriers due to adequacy of mild variant c.79G > A and complex inheritance of other factors such as presence of α-globin gene mutations, mutation in Krüppel-like Factor 1 (KLF1) gene, promoter mutations of gamma-globin gene, iron deficiency anemia, hereditary persistence of fetal Hb etc. [15].

High Resolution Melting (HRM) Analysis is a rapid, high-throughput technique for screening genetic variations in the nucleic acid sequences using the melting properties of double stranded DNA. The HRM analysis enables researchers to rapidly detect and categorize mutations, identify new genetic variants without sequencing. It has been successfully used in detection of mutations in various types of genetic diseases including autosomal recessive, autosomal dominant and X-linked recessive disorders as well as diseases involving somatic mutations [16–20]. Although the HRM analysis has been developed for high throughput screening of mutations in the beta-globin gene in various countries [21, 22], the procedure has not been initiated yet in Bangladesh. The study aimed to devise a rapid mutation screening using HRM curve analysis targeting the hot-spot region. Since the targeted hot-spot harbors most of the common beta-globin gene mutations reported in Bangladesh and other countries of South Asia and Southeast Asia, it could be applied for carrier screening in Bangladesh and some regional countries.

## Methods

### Study participants

A total of 142 transfusion-dependent beta-thalassemia patients in the age range of 1–15 years who came to Dhaka Shisu Hospital (DSH) for follow-up examination for CBC and Hb level followed by blood transfusion were enrolled in the study. The patients had been previously diagnosed with beta-thalassemia by Hb electrophoresis and/or microscopic evaluation of thin blood film. The study also included ninety beta-thalassemia carrier parents and 15 healthy controls who were not thalassemia carriers.

### DNA isolation from whole blood

1 mL of blood was collected in EDTA-coated vacutainer. Genomic DNA (gDNA) was extracted from the collected blood using QIAGEN flexigene® DNA kit (Qiagen, Hilden, Germany) following manufacturer’s instructions.

### Polymerase chain reactions for beta-globin gene amplification

The primers targeting the 428 bp long stretch of HBB gene encompassing the mutational hot-spot region (exon 1, intron 1 and a portion of exon 2) of beta-globin gene in Bangladesh was used for the polymerase chain reactions (PCR) (Table 1) [13, 14]. The PCR amplification was performed in a final reaction volume of 30 μL using 150 ng gDNA as template and the compositions of the PCR components were as follows: 3.0 μL of 10 x PCR buffer (with 1.5 mM MgCl2), 0.9 μL of MgCl2 (25 mM), 6.0 μL of Q-solution (Qiagen), 4.8 μL of dNTP mixture (2.5 mM), 0.6 μL of forward primer (HBB_Ex1F) (10 mM) and 0.6 μL of reverse primer (HBB_Ex2R) (10 mM), 0.3 μL of HotStart Taq DNA polymerase (Qiagen). Finally, the total reaction volume was made 30.0 μL with nuclease-free water. The following thermal cycling profile was used for PCR amplification: initial denaturation at 94 °C for 15 min; 35 cycles of denaturation at 94 °C for 30 s, annealing at 58 °C for 40 s and extension at 72 °C for 40 s; and a final extension at 72 °C for 5 min.

**Table 1:** Primers for mutational hot spot amplification of beta-globin gene

Sl No	Primer name	Primer sequence(5′ → 3′)	Primer size
1	HBB_Ex1F	GGCAGAGCCATCTATTGCTTAC	22
2	HBB_Ex2R	CAGGCCATCACTAAAGGCACC	21

#### Purification of PCR product

Purification of the PCR products was performed using the MinElute® PCR purification kit (Qiagen) following the manufacture’s guidelines.

#### Sequencing of PCR products

Following manufacturer’s instruction, the cycle sequencing of the column-purified PCR product was performed using the Big Dye Version 3.1 Cycle Sequencing Kit (Applied Biosystems, Warrington, UK). Thereafter, the cycle sequencing product was purified using the BigDye® XTerminator™ purification kit (Applied Biosystems) and the manufacturer’s protocol was followed for the purification steps. Finally, the purified cycle sequencing PCR product was subjected to capillary sequencing in an ABI PRISM 310 Automated Sequencer (Applied Biosystems).

#### Sequencing data collection and identification of mutations

The sequencing data were collected using ABI PRISM 310 data collection software version 3.1.0 (Applied Biosystems). The collected data were exported in FASTA format which were then analyzed to identify substitution or deletion mutations by using Basic Local Alignment Search Tool (BLAST), which compared the query sequence with the reference (wild type) sequence (NC_000011.10) retrieved from NCBI database.

#### Real-time PCR-based high resolution melting curve analysis

The real time PCR reactions were performed in a 10 μL reaction volume in a 96-well reaction plate. The PCR mixture contained 5 μL of 2X precision melt supermix (BioRad), 0.2 μL of the forward primer (10 μM), 0.2 μL of the reverse primer (10 μM), 50 ng of genomic DNA in a total of 10 μL reaction volume. The list of primers used for high resolution melt curve analysis is shown in Table 2. The real-time PCR was followed by HRM analysis and the procedures were performed in a CFX96 Touch™ Real-Time PCR machine (BioRad). The real-time PCR with the first set of primers (P1R1) had the following thermal cycling protocol: initial denaturation at 95 °C for 2 min; 40 cycles of denaturation at 94 °C for 10 s, annealing at 60 °C for 15 s and extension at 72 °C for 15 s. Similar to the first set of primers, the thermal cycling protocol for the 2nd set of primers (P2R2) was exactly same except that the annealing temperature for the latter was set to 58^0^c instead of 60^0^c. After the real time PCR was completed, the subsequent melt curve program was identical for both primer sets and it had the following three steps: denaturation at 95 °C for 30 s, renaturation at 60 °C for 30 s, and then melting at 65 °C to 95 °C with an increment of 0.1^0^c per 5 s. After completion of the real time PCR-HRM, the data were analyzed using Precision Melt Analysis™ Software (BioRad). The melt curve shape sensitivity for cluster detection was set to 100%. The difference in Tm threshold for the cluster detection was set to 0.1 and the normalized and the temperature shifted views were used for analysis.

**Table 2:** The list of primers for high resolution melting curve analysis for detection of beta-globin gene mutations

Sl No	Primer name	Primer sequence (5′ → 3′)	Primer size
1	P1	ATGGTGCATCTGACTCCTGAG	21
2	R1	CCAATAGGCAGAGAGAGTCAGTG	23
3	P2	CACTGACTCTCTCTGCCTATTGG	23
4	R2	CAGGCCATCACTAAAGGCACC	21

P1R1 = 1st set of primers for HRM; P2R2 = 2nd set of primers for HRM

## Results

### Selection of mutational hot-spot in the HBB gene

**Fig. 1:**
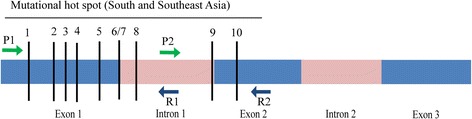
Schematic representation of mutational hot-spot in the beta-globin gene. The blue bars indicate the exons, whereas the pink bars indicate introns. The black horizontal line that starts at c.1 of exon 1 and extends to c.217 of exon 2 comprises the mutational hot-spot of beta-globin gene in South Asia and Southeast Asia. The black vertical lines and the corresponding numbers above the lines represent 10 mutational positions (1 = c.27_28insG, 2 = c.46delT, 3 = c.47G > A, 4 = c.51delC, 5 = c.79G > A, 6/7 = c.92G > C/c.92G > A, 8 = c.92 + 5G > C, 9 = c.92 + 130G > C, and 10 = c.126_129delCTTT) in the HBB gene in Bangladesh. The green and blue arrows indicate the forward primers and the reverse primers respectively that were used for the HRM analysis

Previous studies revealed that most of the beta-globin (HBB) gene mutations (>95%) in Bangladeshi thalassemia patients including three most common mutations, namely c.79G > A, c.92 + 5 G > C and c.126_129delCTTT account for ~85% of the beta-globin gene mutant alleles and these mutations are confined in the exon-1 (c.1 – c.92), the intron-1 (c.92 + 1 – c.92 + 130) and a portion of exon-2 (c.93 – c.217), thus constituting the hot-spot region (Table 3 and Fig. 1). The same hot-spot accounts for over 90% mutations in the HBB gene in India (West Bengal, Uttar Pradesh, Eastern India, and Southern India), Sri Lanka, and Malaysia and over 83%, 82% and 80% of the HBB gene mutations in Thailand, Pakistan, and Mayanmar, respectively [13, 14, 23–28]. Therefore, the HBB gene hot-spot region was targeted for mutation screening by the high resolution melting curve (HRM) analysis. With this view in mind, two sets of primers flanking the hot-spot were designed to analyze beta-thalassemia DNA specimens by the HRM analysis. The first set of primers (Forward P1: c.1 – c.21 and Reverse R1: reverse complement of c.92 + 88 – c.92 + 111) could identify the mutations that were flanked in a portion of exon-1 and intron-1, while the second set of primers (Forward P2: c.92 + 88 – c.92 + 111 and Reverse R2: reverse complement of c.207 – c.228) flanked a portion of intron-1 and exon-2 of the hot-spot region. The interfering effect of the SNP c.9 T > C, which is frequently found in Bangladeshi population, was avoided by using a primer (Forward P1: c.1 – c.21) that could overlay the SNP [29]. As the primer overlay the SNP, it did not become a part of the final PCR product, indicating that the SNP c.9 T > C could not influence the HRM results by its interfering effect. Overall, the primer sets targeting the hot-spot region did have a significance to detect majority of the beta-globin gene mutations.

**Table 3:** The spectrum of HBB gene mutations in the regional hot-spot of South Asia and Southeast Asia

Sl No.	Countries	Mutations	Percentage	References
1	Bangladesh	c.79G > A, c.92 + 5 G > C, c.126_129delCTTT, c.92 G > C, c.27_28insG, c.47G > A, c.92 G > A, c.46delT, c.92 + 130 G > C, and c.51delC	>95%	[13, 14]
2	India: West Bengal, Uttar Pradesh, Eastern India, Southern India	c.92 + 5 G > C, c.92 + 1G > C, c.27_28insG, c.47G > A, c.51delC, c.92 + 5 G > C, c.126_129delCTTT	> 90%	[23]
3	Sri Lanka	c.92 + 5 G > C, c.92 + 1G > C, c.79G > A, c.92 G > C, c.27_28insG, c.47G > A, c.51delC, c.126_129delCTTT	>90%	[24]
4	Malaysia	c.92 + 5 G > C, c.92 + 1 G > C, c.59A > G, c.126_129delCTTT, c.52A > T, c.27_28insG, c.216_217insA, c.92 + 1 G > C, c.79G > A	>90%	[25]
5	Thailand	c.126_129delCTTT, c.52A > T, c.59A > G, c.27_28insG, c.92 + 1 G > C, c.92 + 5 G > C, c.108C > A, c.47G > A	>83%	[26]
6	Pakistan	c.92 + 5 G > C, c.27_28insG, c.92 + 1G > C, c.126_129delCTTT, c.92 G > A, c.17_18delCT and c.47G > A	>82%	[27]
7	Mayanmar	c.92 + 1 G > T, c.126_129delCTTT, c.92 + 5 G > C, c.53A > T, c.135delC, c.108C > A, c.47G > A, c.51delC, c.46delT, c.92 + 1 G > C, c.27_28insG, c.126delC	>80%	[28]

### Selection of reference samples with known mutations by sequencing of beta-thalassemia DNA specimens targeting the HBB gene hot-spot

Targeting the hot-spot region of beta-globin gene, a total of 101 beta-thalassemia DNA specimens were subjected to Sanger sequencing. The DNA sequencing data demonstrated that 98% (99/101) of the specimens had either compound heterozygous mutations or homozygous mutations and 2% (2/101) had heterozygous mutations within the hot-spot, indicating the validity of the use of the hot-spot to detect the beta-globin gene mutations in Bangladesh. Accordingly, we could identify a total of twelve different mutations; namely c.27_28insG, c.33C > A, c.46delT, c.47G > A, c.51delC, c.79G > A, c.92G > C, c.92 + 5G > C, c.92 + 130G > C, c.126delC, c.126_129delCTTT and c.135delC. The combination patterns of mutations which had been identified as the genotypes of the recessive disorder are presented in Table 4. Among the above-mentioned 12 mutations, c.33C > A, c.126delC, and c.135delC mutations had not been reported previously for the Bangladeshi population. Based on the mutational information, 40 participants who were parents of beta-thalassemia patients were recruited and their DNA specimens were sequenced to get samples with known heterozygous mutations (Additional file 1: Table S1). Parents of beta-thalassemia participants with rare compound heterozygous mutations c.33C > A and c.51delC were not available in the study. The specimens with known mutations, as manifested by DNA sequencing of beta-thalassemia patients and their parents, were used than for initial evaluation of HRM approach for heterozygous mutations screening, which targeted 10 mutations namely, c.27_28insG, c.46delT, c.47G > A, c.79G > A, c.92G > C, c.92 + 5G > C, c.92 + 130G > C, c.126delC, c.126_129delCTTT and c.135delC of the beta-globin gene.

**Table 4:** The sequence-based identification of combinations of mutations in the beta-thalassemia specimens and corresponding HRM primer sets

Sl no.	Combinations of mutations	Number of samples	Primers for HRM
1	c.79 G > A^a^ and c.92 + 5 G > C^a^	55	P1R1
2	c.92 + 5 G > C^b^	21	P1R1
3	c.47G > A^a^ and c.79G > A^a^	3	P1R1
4	c.79 G > A^a^ and c.126_129delCTTT^a^	3	P1R1& P2R2
5	c.46delT^a^ and c.79G > A^a^	2	P1R1
6	c.27-28insG^a^ and c.79G > A^a^	2	P1R1
7	c.92 G > C^a^ and c.92 + 5 G > C^a^	2	P1R1
8	c.79G > A^a^ and c.92G > C^a^	2	P1R1
9	c.92 + 5 G > C^a^ and c.92 + 130 G > C^a^	2	P1R1 & P2R2
10	c.92 + 5 G > C^a^ and c.126_129delCTTT^a^	2	P1R1 & P2R2
11	c.79G > A^b^	1	P1R1
12	c.79G > A^a^	1	P1R1
13	c.79G > A^a^ and c.92 + 130 G > C^a^	1	P1R1 & P2R2
14	c.33C > A^a^ and c.51delC^a^	1	P1R1
15	c.79G > A^a^ and c.126delC^a^	1	P1R1 & P2R2
16	c.126_129delCTTT^b^	1	P2R2
17	c.135delC^a^	1	P2R2

^a^ = Indicates heterozygous mutation; ^b^ = indicates homozygous mutations

Furthermore, as a means of cost-saving approach, sixteen out of 101 beta-thalassemia specimens with sequence-based known mutations were selected as references for HRM analysis to see whether the approach could perform direct genotyping of beta-thalassemia patients. The references were sufficient to represent all of the 12 mutations and their common compound heterozygous combination patterns because three reference samples had homozygous c.79G > A, c.92 + 5 G > C, and c.126_129delCTTT mutations and 12 had compound heterozygous mutations, namely c.79G > A plus c.92 + 5G > C, c.47G > A plus c.79G > A, c.79G > A plus c.126_129delCTTT, c.46delT plus c.79G > A, c.27-28insG plus c.79G > A, c.92G > C plus c.92 + 5G > C, c.79G > A plus c.92G > C, c.92 + 5G > C plus c.92 + 130G > C, c.92 + 5G > C plus c.126_129delCTTT, c.79G > A plus c.92 + 130G > C, c.33C > A plus c.51delC, and c.79G > A plus c.126delC and a sample had heterozygous c.135delC deletion.

### Screening of heterozygous beta-globin gene mutations by HRM

**Fig. 2:**
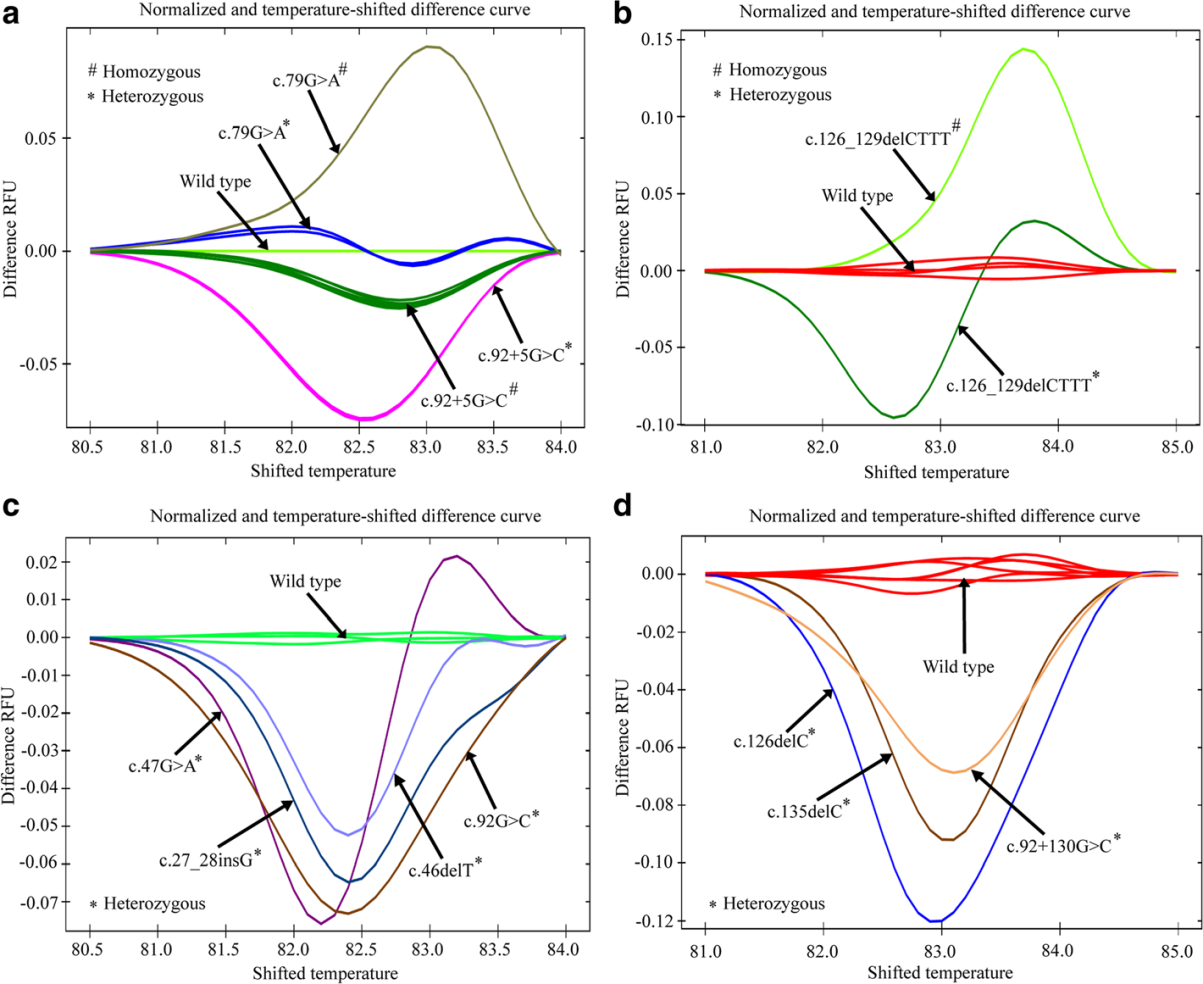
HRM-based screening of HBB gene mutations. **a** Normalized and temperature shifted difference curve patterns of c.79G > A and c.92 + 5G > C, (**b**) Normalized and temperature shifted difference curve patterns of c.126_129delCTTT, (**c**) Normalized and temperature shifted difference curve patterns of c.27_28insG, c.46delT, c.47G > A and c.92G > C, and (**d**) Normalized and temperature shifted difference curve patterns of c.92 + 130G > C, c.126delC and c.135delC alleles. The curve shape generated by each mutant allele was compared to its wild type counterpart and other mutant alleles. * indicates heterozygous alleles and # indicates homozygous alleles

False negative results due to the presence of mild allele such as c.79G > A is a big concern in the mean corpuscular volume (MCV) ≤80 fL based screening of beta-thalassemia carrier. Furthermore, the electrophoretic parameter HbA2-dependent screening may also give rise to false negative or false positive results based on the coinheritance of the α-globin and Krüppel-like Factor 1 (KLF1) genes mutations, the gamma-globin gene promoter mutations, and iron deficiency anemia. Thus, the establishment of HRM-based screening might be useful for carrier screening to supplement the hematological and electrophoretic indices. Previous studies have demonstrated that c.79G > A, c.92 + 5G > C and c.126_129delCTTT mutations account for 85% of beta-globin related complications. The pre-genotyped specimens were subjected to HRM analysis. The normalized melt curves for c.79G > A, and c.92 + 5G > C mutations are shown in Additional file 2: Figure S1; whereas the normalized melt curves for c.126_129delCTTT mutation are shown in Additional file 3: Figure S2. The temperature shifted difference curves of these three HBB gene mutations are shown in Fig. 2. The difference curves generated by the heterozygous and homozygous alleles could be easily distinguished from the wild type samples based on differences in the melting curve shapes. As shown in Fig. 2a, the difference in relative fluorescence unit (RFU) for homozygous c.79G > A allele was always higher than that for the wild type allele. However, the difference in RFU for heterozygous c.79G > A allele was initially higher, which then dropped below the wild type allele and then finally raised again above the wild type allele. For the homozygous and heterozygous c.92 + 5G > C alleles, the difference in RFU was always lower than that for the wild type allele. However, the heterozygous c.92 + 5G > C allele had a greater reduction in fluorescence intensity compared to the homozygous c.92 + 5G > C allele (Fig. 2a). The allele with deletion mutation c.126_129delCTTT in homozygous state could produce a melt curve that always had a higher fluorescence intensity compared to its wild type counterpart throughout the melting procedure. In contrast, the heterozygous c.126_129delCTTT allele could generate a melt curve with higher fluorescence intensity in the later stages of melting compared to the wild type allele (Fig. 2b).

Moreover, other less frequent heterozygous mutations including c.27_28insG, c.46delT, c.47G > A, c.92G > C and c.92 + 130G > C, and previously unreported mutations in Bangladesh, namely c.126delC and c.135delC were also distinguishable from their respective wild type alleles (Fig. 2c, Fig. 2d, Additional file 4: Figure S3 and Additional file 5: Figure S4). As presented by the normalized and temperature shifted difference curves (Fig. 2c, and Fig. 2d), all of these heterozygous mutational statuses showed a weaker fluorescence intensity during melting compared to their wild type counterparts except c.47G > A, which showed a lower fluorescence intensity during the initial phases of the melting and a higher fluorescence intensity in the latter phases.

After initial testing of pre-genotyped samples, 50 specimens from the parents of beta-thalassemia patients were analyzed without any prior knowledge of their genotypes to validate the assay. All of the specimens had sequence variations from their respective wild type alleles. The sequencing data revealed that 29 out of 50 specimens had c.92 + 5G > C, 18 had c.79G > A, 2 had c.126_129delCTTT and one had c.126delC heterozygous states, respectively (Additional file 6: Figure S5 and Additional file 7: Figure S6). It was noticeable that all c.92 + 5G > C heterozygotes together formed a cluster. Similarly, c.79G > A and c.126_129delCTTT heterozygotes also formed their respective clusters. One c.126delC formed its distinct melt curve which was distinguishable from other heterozygotes. The significance of the approach lies in the fact that it could be applied for mass carrier screening for prevention and control of beta-thalassemia.

### Compound heterozygous mutations could be distinguished from wild type alleles by HRM

**Fig. 3:**
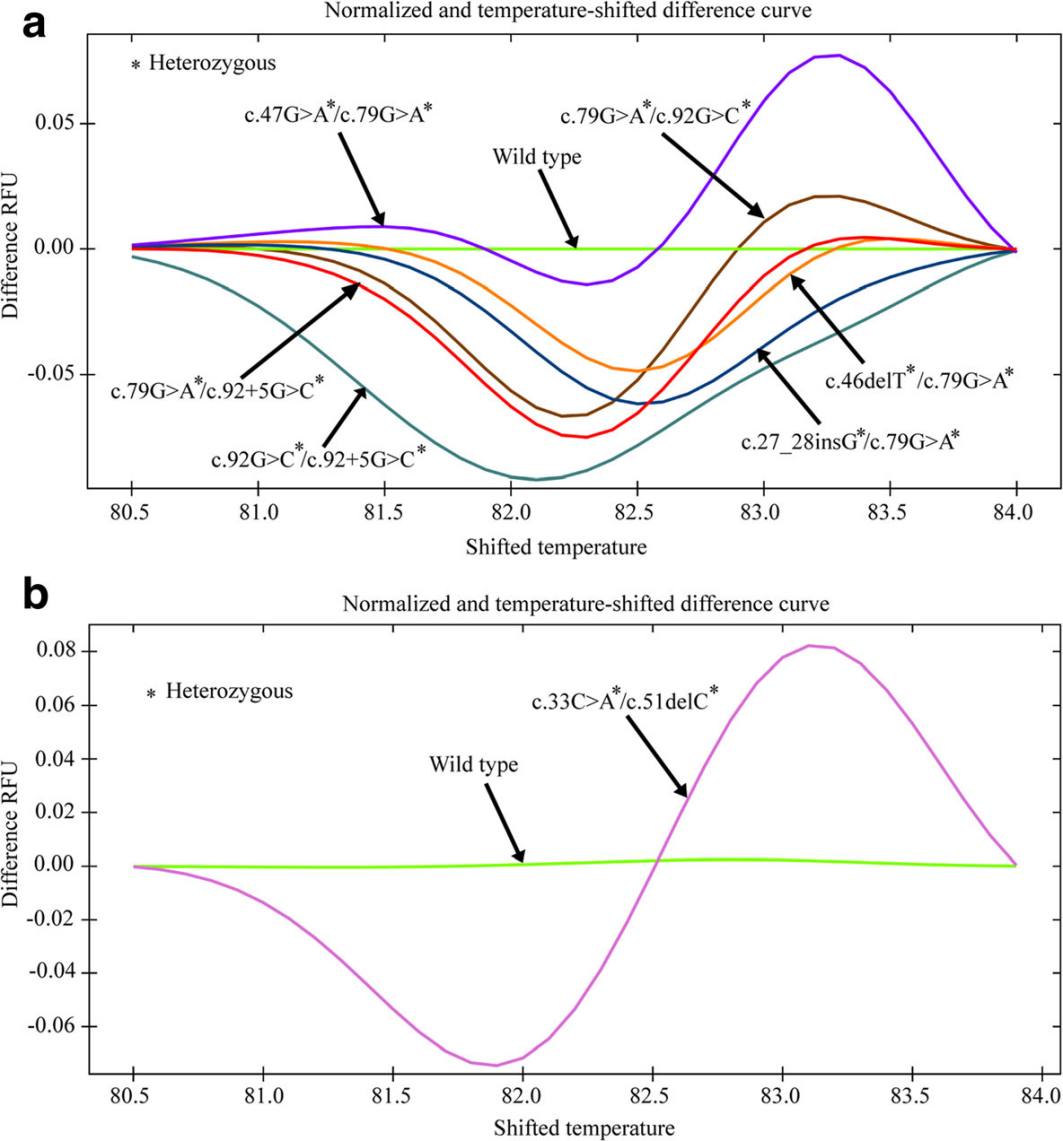
HRM curves patterns for the compound heterozygous mutations. **a** The normalized and temperature shifted difference curves for the compound heterozygous mutations c.47G > A plus c.79G > A, c.79G > A plus c.92G > C, c.46delT plus c.79G > A, c.27_28insG plus c.79G > A, c.79G > A plus c.92 + 5G > C, c.92G > C plus c.92 + 5G > C and wild type alleles. **b** The normalized and temperature shifted difference curves for the rare compound heterozygous mutations c.33C > A plus c.51delC and wild type allele

Our data have already demonstrated that both common as well as rare mutations could be screened using HRM. Next, as a means of cost-saving approach, we wanted to observe whether the compound heterozygous mutations could be separated from their respective wild type alleles for direct genotyping of beta-thalassemia patients. Mutations in the HBB gene other than in the positions c.79 and c.92 + 5 usually occur as co-existing partners either with c.79G > A or c.92 + 5G > C in beta-thalassemia patients. Most of the other HBB gene mutations of beta-thalassemia patients had been detected in close proximity to the common mutations, namely c.79 G > A and c.92 + 5 G > C, suggesting that the compound heterozygous mutations could be screened using a single pair of primers. The compound heterozygous mutations including c.47G > A plus c.79G > A, c.79G > A plus c.92G > C, c.46delT plus c.79G > A, c.27_28insG plus c.79G > A, c.79G > A plus c.92 + 5 G > C, and c.92G > C plus c.92 + 5 G > C generated curves that were distinguishable from the wild type alleles (Fig. 3a). The dsDNA melting patterns generated by combination mutation partners, namely 46delT plus c.79G > A, c.27_28insG plus c.79G > A, c.79G > A plus c.92 + 5 G > C, and c.92G > C plus c.92 + 5 G > C could be unambiguously distinguished from the wild type alleles due to a significant reduction in RFU during melting. On the other hand, the compound heterozygous mutations c.79G > A plus c.92G > C generated a melt curve that had a relatively shorter plateau followed by a longer curved path, which then reached the peak above the wild type RFU path. Moreover, the melting curve pattern generated by c.47G > A plus c.79G > A was more diverse than any other combinations of mutations. It produced the longest plateau in the beginning and then followed the curved path below the wild type allele and finally reached the peak point which lay above the path of wild type allele.

The rare compound heterozygous mutations c.33C > A plus c.51delC could also be distinguished from the wild type allele due to its distinctive melting pattern that had a relatively lower fluorescence intensity in the first half of the melting and had a higher fluorescence intensity in the second half of the melting compared to its wild type counterpart (Fig. 3b). The findings indicate that the compound heterozygous mutations could be screened by HRM.

### Direct genotyping of unknown beta-thalassemia samples using HRM

**Fig. 4:**
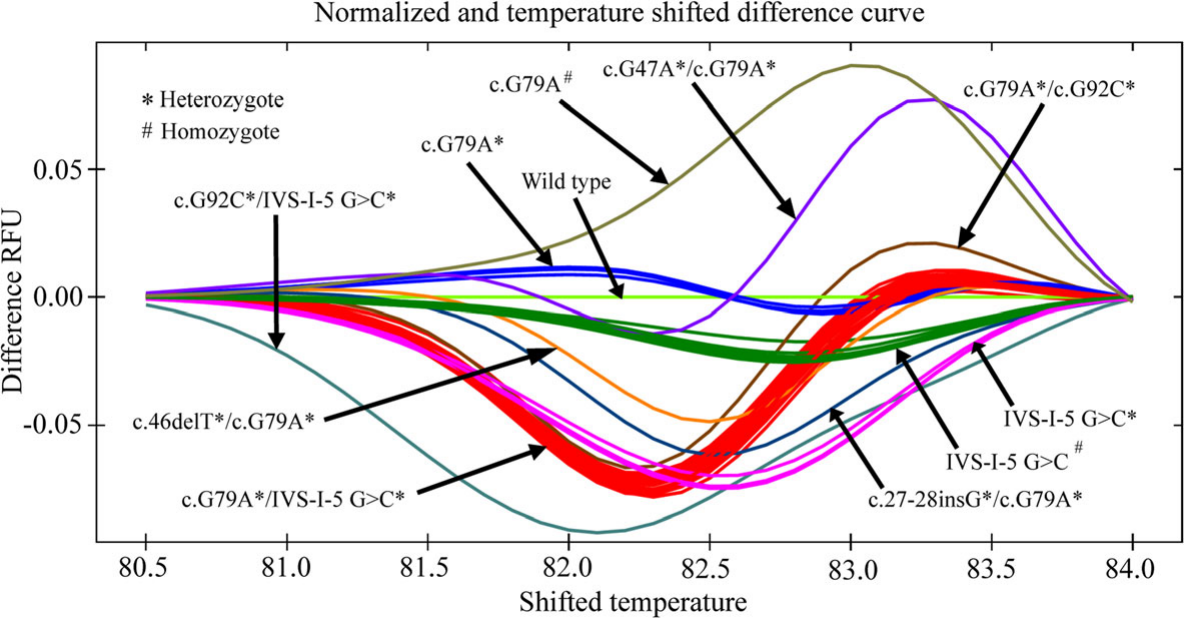
HRM curve patterns for unknown samples using 1st set of primers (P1R1) covering exon-1 and a portion of intron-1. The normalized and temperature shifted difference curve could separate various combinations of mutations including homozygous (#), heterozygous (*) or compound heterozygous (*/*) states from wild type alleles and from each other

**Fig. 5:**
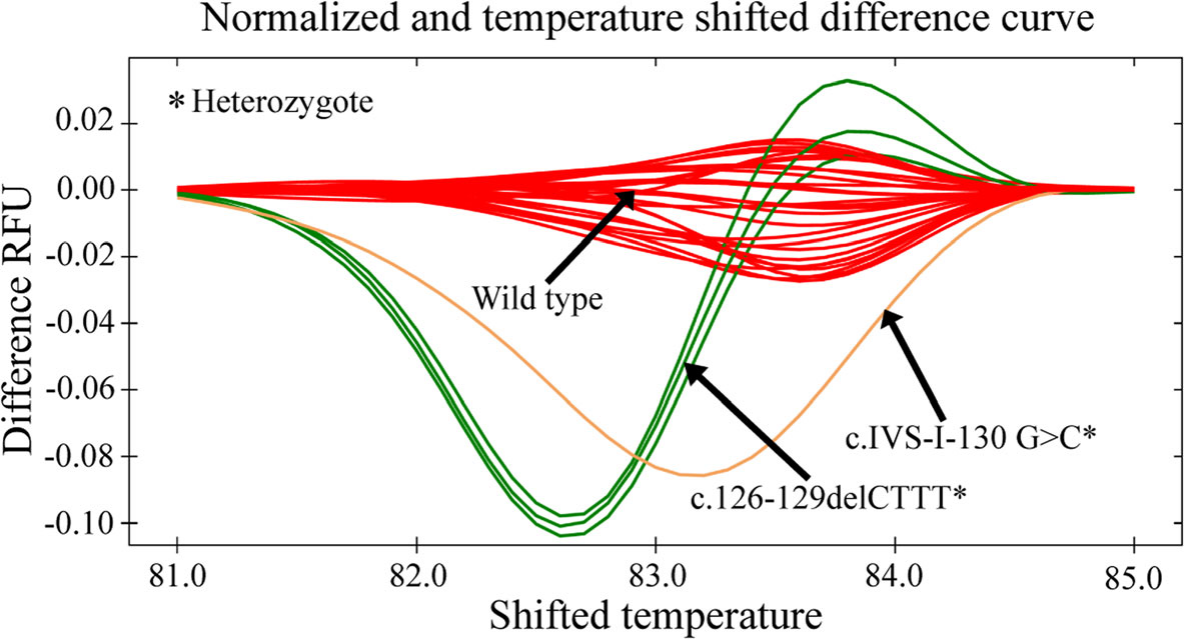
The HRM curve analysis for unknown samples using the 2nd set of primers (P2R2) covering a portion of intron-1 and exon-2. The normalized and temperature shifted difference curve could separate various mutations from the wild type alleles and from each other

The HRM curve analysis with reference samples of known mutations has already demonstrated that HRM could be a very good approach for distinguishing different types of mutations from the wild type alleles. Combinations of mutations could also be distinguished from one another by HRM. The findings prompted us to investigate whether the HRM approach could be applied for genotyping of beta-thalassemia samples for the confirmation of the disease status. In order to test this hypothesis, we performed HRM analysis on 41 unknown samples without any prior knowledge of their mutational status. The reference samples with known mutations were also run side-by-side to calibrate the data. All of these forty one samples differed in HRM curve patterns from the control wild type alleles. Each one of them had exhibited melting patterns similar to that of the corresponding reference samples with known homozygous, heterozygous or compound heterozygous mutations (Fig. 4, Fig. 5 and Table 5). Among forty one unknown samples, twenty three specimens generated the melt curves similar to one generated by the reference sample having compound heterozygous c.79G > A plus c.92 + 5G > C mutations, eight specimens produced the melt curves that resembled one generated by the reference sample with homozygous c.92 + 5G > C mutation, and two samples generated the melt curves that resembled the melt curve produced by the reference sample having compound heterozygous mutation c.79G > A plus c.126_129delCTTT. Each of the remaining eight unknown samples generated a single melt curve that resembled the melt curve produced by reference samples with the following homozygous mutation c.79G > A and compound heterozygous mutations c.27-28insG plus c.79G > A, c.47G > A plus c.79G > A, c.92 + 5 G > C plusc.92 + 130 G > C, c.92 + 5 G > C plus c.126_129delCTTT, c.79G > A plus c.92G > C, c.46delT plus c.79G > A, and c.92G > C plusc.92 + 5 G > C, respectively. The DNA sequencing data of the unknown samples also validated the HRM result (Table 5).

**Table 5:** Combinations of mutations identified by HRM and confirmed by sequencing of unknown samples

Sl no.	Combinations of mutations	Number of samples	Primers for HRM
1	c.79G > A^a^ and c.92 + 5 G > C^a^	23	P1R1
2	c.92 + 5 G > C^b^	8	P1R1
3	c.79G > A^a^ and c.126_129delCTTT^a^	2	P1R1 & P2R2
4	c.79G > A^b^	1	P1R1
5	c.27-28insG^a^ and c.79G > A^a^	1	P1R1
6	c.47G > A^a^ and c.79G > A^a^	1	P1R1
7	c.92 + 5G > C^a^ and c.92 + 130 G > C^a^	1	P1R1 & P2R2
8	c.92 + 5G > C^a^ and c.126_129delCTTT^a^	1	P1R1 & P2R2
9	c.79G > A^a^ and c.92G > C^a^	1	P1R1
10	c.46delT^a^ and c.79G > A^a^	1	P1R1
11	c.92G > C^a^ and c.92 + 5G > C^a^	1	P1R1

^a^ = Indicates heterozygous mutation; ^b^ = indicates homozygous mutations

In summary, the HRM approach based on mutational hot-spot could be useful for screening of carriers as well as disease status of beta-thalassemia in Bangladesh. There might have new HRM patterns other than the references and in such cases the samples would require sequencing for identification of the mutations.

## Discussion

Inherited disorders of Haemoglobin make up the most commonly observed genetic diseases, resulting in significant morbidity and mortality worldwide. It has been reported that between 300,000 to 400,000 children are born with severe hemoglobinopathies each year, and among them 95% are affected with abnormalities of the β-globin gene (*HBB*) [1]. A study conducted on school children in Bangladesh demonstrated that the beta-thalassemia and hemoglobin E carrier statuses were 4.1% and 6.1% respectively [30], indicating that as many as 8990 thalassemia babies are born each year in Bangladesh with its population of about 160 million and a birth rate of 2.16%, as manifested by Hardy-Weinberg equation. Now, it has been agreed upon at all levels of public and private policy-making to create general awareness and adopt appropriate preventive and control measurements for reduction of beta-thalassemia in Bangladesh. Adoption of mass carrier screening, especially premarital and prenatal screening for carrier detection is required for proper counseling and goal-oriented motivation. In 1970s, the premarital screening programs were initiated in Greece and Cyprus and these proved to be huge successes in decreasing the total number of children being born with β-thalassaemia [31, 32]. Then a number of countries where beta-thalassemia had high prevalence, adopted an approach similar to that of Greece and Cyprus in pursuit of beta-thalassemia prevention [33]. The success of such premarital screening programs depends on the rapidity, reliability and cost-effectiveness of the procedures used to detect the carriers. The conventional strategy for thalassemia screening includes (a) analysis of blood specimens for CBC, where MCV value ≤80 fL indicates thalassemia carriers, (b) analysis of electrophoresis indices for specimens of the suspected carriers, where a specimen with HbA2 > 3.5 is highly considered as a thalassemia carrier, and (c) testing of specimens with HbA2 > 3.5 at the DNA level, where a mutation in any globin gene confirms the carrier status. Each step of the carrier screening has some limitations and we opted to use HRM to overcome those barriers to supplement the conventional methodologies for thalassemia screening [15]. Needless to say, the present approach focused only on beta-thalassemia because it represents the major concerns regarding hemoglobinopathy-related burdens in Bangladesh.

The study has demonstrated that clinically silent or mild mutation c.79G > A could be screened successfully using HRM analysis. This is really important for prevention of beta-thalassemia as the MCV-based screening approach may miss a significant number of E-traits [15]. This is because c.79G > A mutation might not always mean that MCV will be below 80 fL in E-trait. Therefore, if a specimen with MCV ≥ 80 fL is not subjected to further assessment, there is a chance that an E-trait could be missed. The clinically silent c.79G > A mutation is common not only in Bangladesh [13, 14] but also in other South Asian and Southeast Asian countries [34–37]. It is mentionable here that c.79G > A mutation can exert serious outcomes when it becomes a partner with other severe beta-globin gene mutations, such as c.92 + 5G > C in a compound heterozygous state, which may produce a condition that mimics the β-thalassemia major [34, 38]. Thus the screening which is based exclusively on MCV might not be well suited for preventative measures in Bangladesh and other South Asian and Southeast Asian countries as c.79G > A is a very common mutant HBB gene allele in these regions; indicating that the required screening approach must be a robust, reliable, high throughput and easy-to-perform method like HRM.

In addition to the silent c.79G > A mutation, other common mutations including c.92 + 5G > C and c.126_129delCTTT as well as less frequent heterozygous mutations like c.27_28insG, c.46delT, c.47G > A, c.92G > C, c.92 + 130G > C, c.126delC, and c.135delC were differentiated successfully by our HRM approach. The established HRM method can also supplement the second step of carrier screening approach which is based on hemoglobin A2 quantification. HbA2 quantification is widely used for carrier screening [39]. However, β-globin gene mutations which result in only minimal elevation in HbA2 levels could be missed easily during screening [40]. Moreover, external conditions that influence HbA2 level are known to complicate the interpretation of β-thalassemia screening results. These external conditions include (a) α-thalassemia and iron deficiency anemia which may have influence on lowering HbA2 levels and (b) mutations in the Krüppel-like Factor 1 (KLF1) gene which is known to associate with elevation in HbA2 level [41–43]. Additionally, co-inheritance of mutations in β-globin and δ-globin genes (*HBD*) could lead to HbA2 levels that are normal or even reduced [44]. Consequently, even though the quantification of HbA2 is a useful screening tool, use of HRM could be a supplemental approach that could overcome the limitations of HbA2 quantification only.

Although other DNA-based screening approaches including Denaturing High Performance Liquid Chromatography (DHPLC), Single Strand Conformational Polymorphism (SSCP) and Denaturing Gradient Gel Electrophoresis (DGGE) could provide alternative means of detection of point mutations and small deletions in the beta-globin gene, each of these approaches has some limitations that include technical difficulties, requirement of post amplification downstream processes etc. On the other hand, although the Amplification-Refractory Mutation System (ARMS) PCR has the advantage of being a screening method that can detect several mutations simultaneously, it would require two primers sets for each mutation; one set for the mutant allele and another set for the wild type allele, and in addition, it requires an internal control, thus making the process complicated, time-consuming and costly. Even detection of adjacent mutations would require multiple primers and controls. Moreover, requirement of downstream processes such as gel electrophoresis and the limitation of being able to detect only specified mutations narrow down its scope. On the other hand, gene scanning by High Resolution Melting curve analysis offers advantages of being a closed tube system with no requirement of complicated post amplification processing by chromatography or gel electrophoresis. Moreover, the adjacent mutations could be screened using only one set of primers and even unknown mutations in the amplified region could be distinguished from the wild type alleles. Thus the HRM approach offers numerous advantages over other available methods for screening of mutations.

The present study differs from other HRM-based studies for HBB gene mutation detection in respect of primer designing. The other studies focused on mutation screening by scanning full length HBB gene by HRM using at least one primer pair per 70 bp–200 bp [21, 29], whereas the current study focused only on the designated mutational hot-spot and only two sets of primers were enough to achieve the goal. This study also emphasizes on the application of the approach in the regional countries of South Asia and Southeast Asia using the same mutational hot-spot (Table 3) provided that this may require additional one or two sets of primers to cover other mutations which may have high prevalence in the regional countries. Above all, the approach may draw attention for its higher sensitivity and specificity as well as its rapidity and reliability.

The HRM-PCR profiling of the amplified products not only pointed out the presence of nucleotide variation but also demonstrated that the exact nucleotide identification is possible when the appropriate reference controls are run side-by-side with the samples. Our HRM approach could detect all variant sequences for all 41 blindly-analyzed samples. Thus, direct genotyping of the samples would be unambiguously identified in the presence of appropriate controls including positive references such as genomic DNA from the confirmed cases with the mutant alleles and wild type controls in the reaction systems. However, samples that are different from our reference controls, having new combination with c.79G > A or c.92 + 5G > C or new compound heterozygous combinations are expected to result in different melt curves. Only those samples with new melting curve patterns would require sequencing to identify their genotypes. It can be assumed that the number of such samples would be very few, thus making the genotyping easy, rapid and inexpensive.

In our laboratory settings, it cost $145.00 ($3.50 per sample) for HRM-based screening of forty one samples by targeting the HBB gene hot-spot using two sets of primers that flanked all the major mutations and even some rare mutations. On the other hand, the cost of ARMS PCR for forty one samples was $125.00 ($3.00 per sample) when only one mutation was targeted, indicating that the cost would be much higher for screening of multiple mutations by ARMS PCR than that of the HRM. Finally, detection of mutations by Sanger Sequencing required approximately $565.00 for forty one samples ($13.75 per sample) which was four times higher than that of HRM-based screening. Thus, on the one hand, the HRM-based screening of mutations is a rapid, reliable and high throughput approach, and, on the other hand, it is less expensive than other genotyping approaches.

## Conclusion

In conclusion, the established HRM approach could be useful for screening of beta-thalassemia trait and hemoglobin E trait targeting the mutational hot-spot of beta-globin gene in Bangladesh and regional countries. It can be a useful supplement of hematological and electrophoretic indices to avoid false positive or false negative results. The approach should deserve attention for nationwide beta-thalassemia carrier screening for its reliability, accuracy, cost-effectiveness, time-saving, and high-throughput operational platform.

## Additional files

Additional file 1: Table S1. Mutational status of 40 parents of beta-thalassemia patients recruited for initial evaluation of HRM study. (DOCX 11 kb)

Additional file 2: Figure S1. Normalized melt curve patterns of homozygous and heterozygous c.79G > A and c.92 + 5G > C mutations. (TIFF 232 kb)

Additional file 3: Figure S2. Normalized melt curve patterns of homozygous and heterozygous c.126_129delCTTT mutations. (TIFF 173 kb)

Additional file 4: Figure S3. Normalized melt curve patterns of heterozygous c.27_28insG, c.46delT, c.47G > A and c.92G > C mutations. (TIFF 211 kb)

Additional file 5: Figure S4. Normalized melt curve patterns of heterozygous c.92 + 130G > C, c.126delC and c.135delC mutations. (TIFF 189 kb)

Additional file 6: Figure S5. Temperature shifted difference curves of unknown carrier parents subjected to HRM analysis by 1st set of primers. (TIFF 530 kb)

Additional file 7: Figure S6. Temperature shifted difference curves of unknown carrier parents subjected to HRM analysis by 2nd set of primers. (TIFF 487 kb)

## Abbreviations

ARMS: Amplification-Refractory Mutation System
DGGE: Denaturing Gradient Gel Electrophoresis
DHPLC: Denaturing High Performance Liquid Chromatography
*HBB*: β-globin gene
*HBD*: δ-globin genes
HRM: High resolution melting
KLF1: Krüppel-like Factor
SSCP: Single Strand Conformational Polymorphism
