# Supplementary material for: High resolution melting curve analysis targeting the HBB gene mutational hot-spot offers a reliable screening approach for all common as well as most of the rare beta-globin gene mutations in Bangladesh
Source: BMC Genet. 2018 Jan 2;19:1. doi: 10.1186/s12863-017-0594-3 (PMC5751541; doi:10.1186/s12863-017-0594-3)
Supplement: Supplementary file 1 — Mutational status of 40 parents of beta-thalassemia patients recruited for initial evaluation of HRM study. (DOCX 11 kb) [file 12863_2017_594_MOESM1_ESM.docx]

**Table S1. Mutational status of 40 parents of beta-thalassemia patients recruited for initial evaluation of HRM study**

| Sl no. | Mutational status | Number of carrier parents |
| --- | --- | --- |
| 1 | c.27_28insG^*^ | 2 |
| 2 | c.46delT^*^ | 2 |
| 3 | c.47G>A^*^ | 2 |
| 4 | c.79G>A^*^ | 10 |
| 5 | c.92G>C^*^ | 2 |
| 6 | c.92+5G>C^*^ | 12 |
| 7 | c.92+130G>C^*^ | 3 |
| 8 | c.126delC^*^ | 1 |
| 9 | c.126_129delCTTT^*^ | 5 |
| 10 | c.135delC^*^ | 1 |
|  |  | Total = 40 |

*Heterozygous for the mutation
