# Supplementary figures and images for: High resolution melting curve analysis targeting the HBB gene mutational hot-spot offers a reliable screening approach for all common as well as most of the rare beta-globin gene mutations in Bangladesh
Source: BMC Genet. 2018 Jan 2;19:1. doi: 10.1186/s12863-017-0594-3 (PMC5751541; doi:10.1186/s12863-017-0594-3)

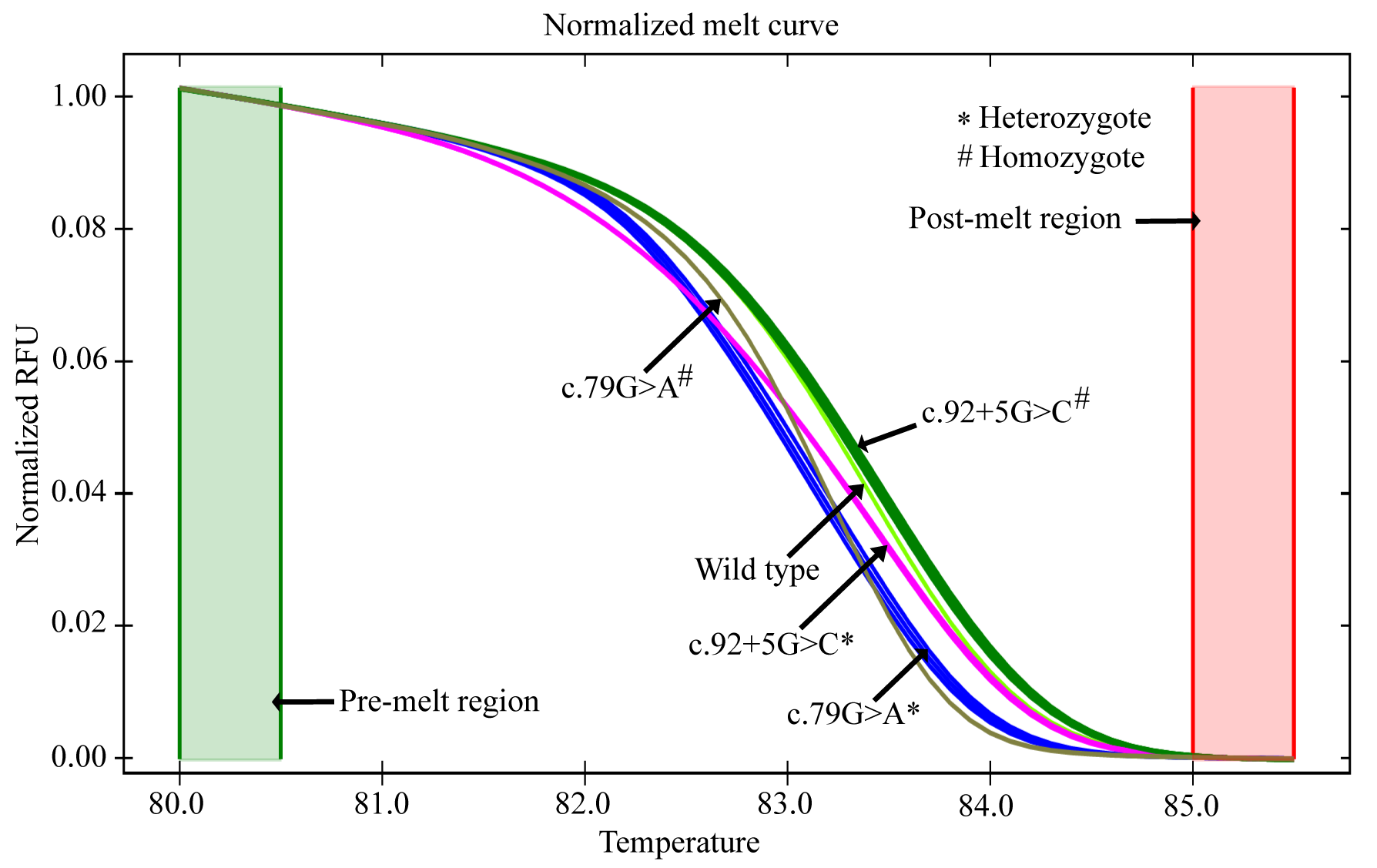

Supplement: Supplementary file 2 — Normalized melt curve patterns of homozygous and heterozygous c.79G > A and c.92 + 5G > C mutations. (TIFF 232 kb) [file 12863_2017_594_MOESM2_ESM.tif]

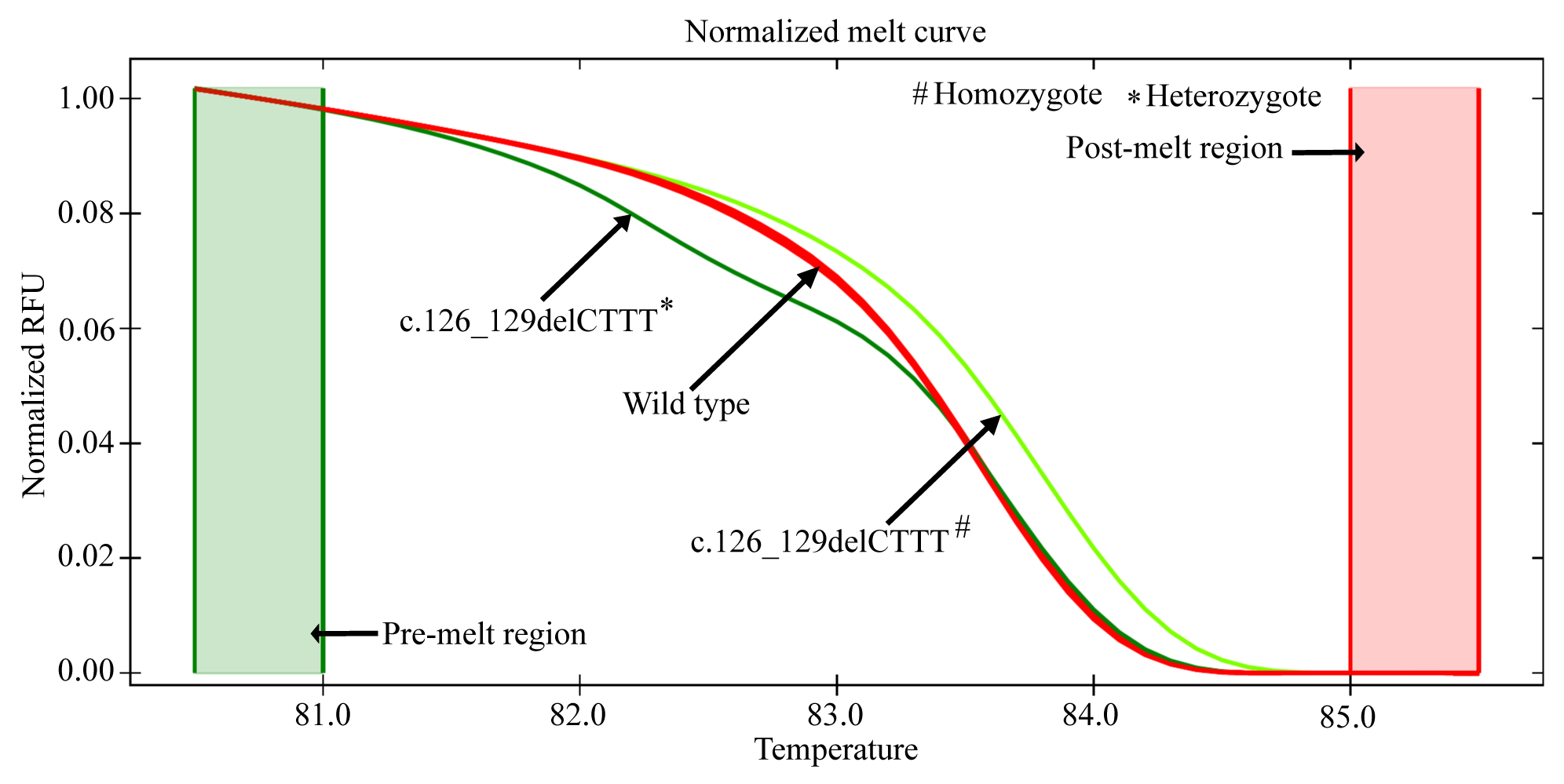

Supplement: Supplementary file 3 — Normalized melt curve patterns of homozygous and heterozygous c.126_129delCTTT mutations. (TIFF 173 kb) [file 12863_2017_594_MOESM3_ESM.tif]

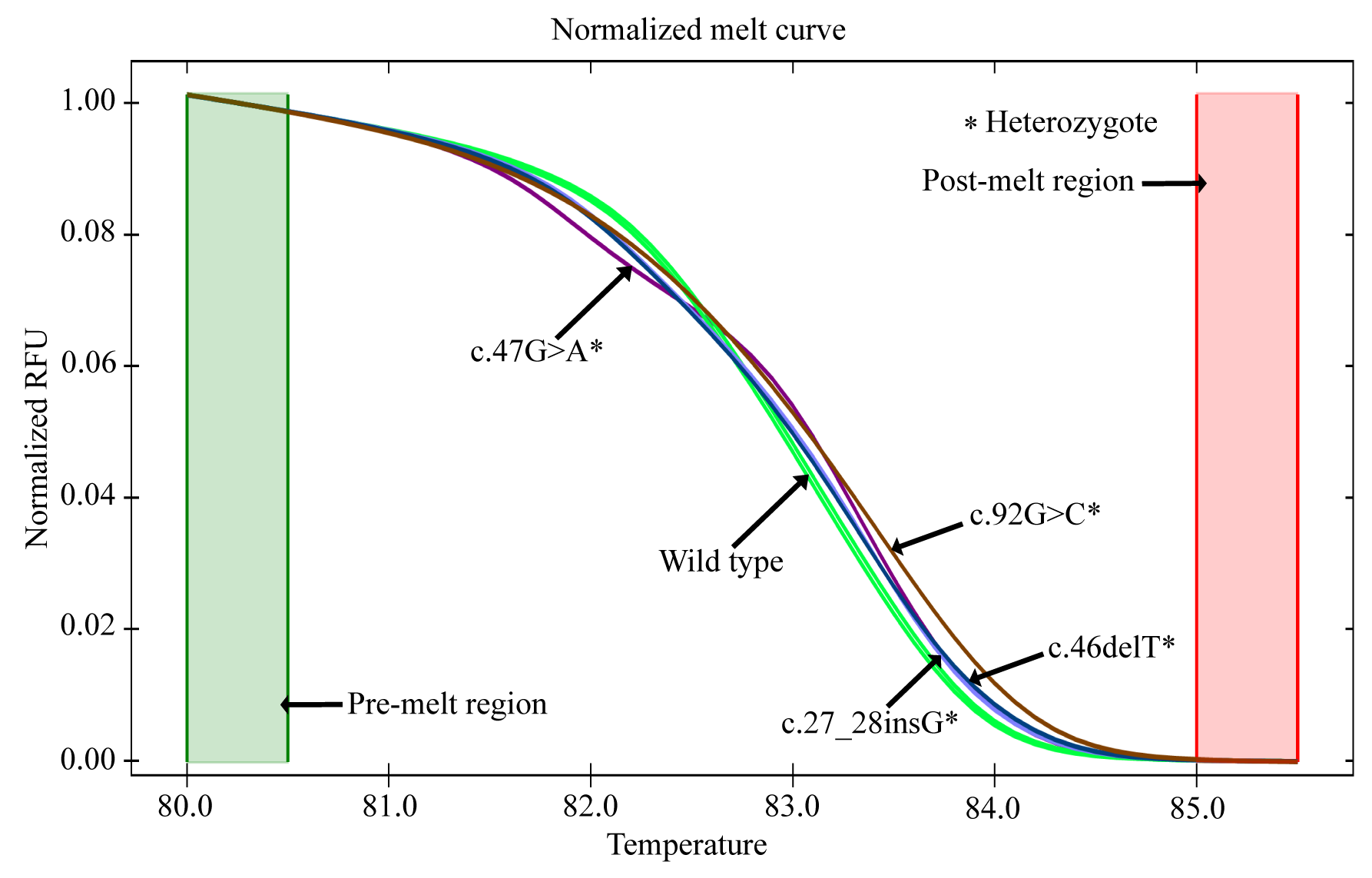

Supplement: Supplementary file 4 — Normalized melt curve patterns of heterozygous c.27_28insG, c.46delT, c.47G > A and c.92G > C mutations. (TIFF 211 kb) [file 12863_2017_594_MOESM4_ESM.tif]

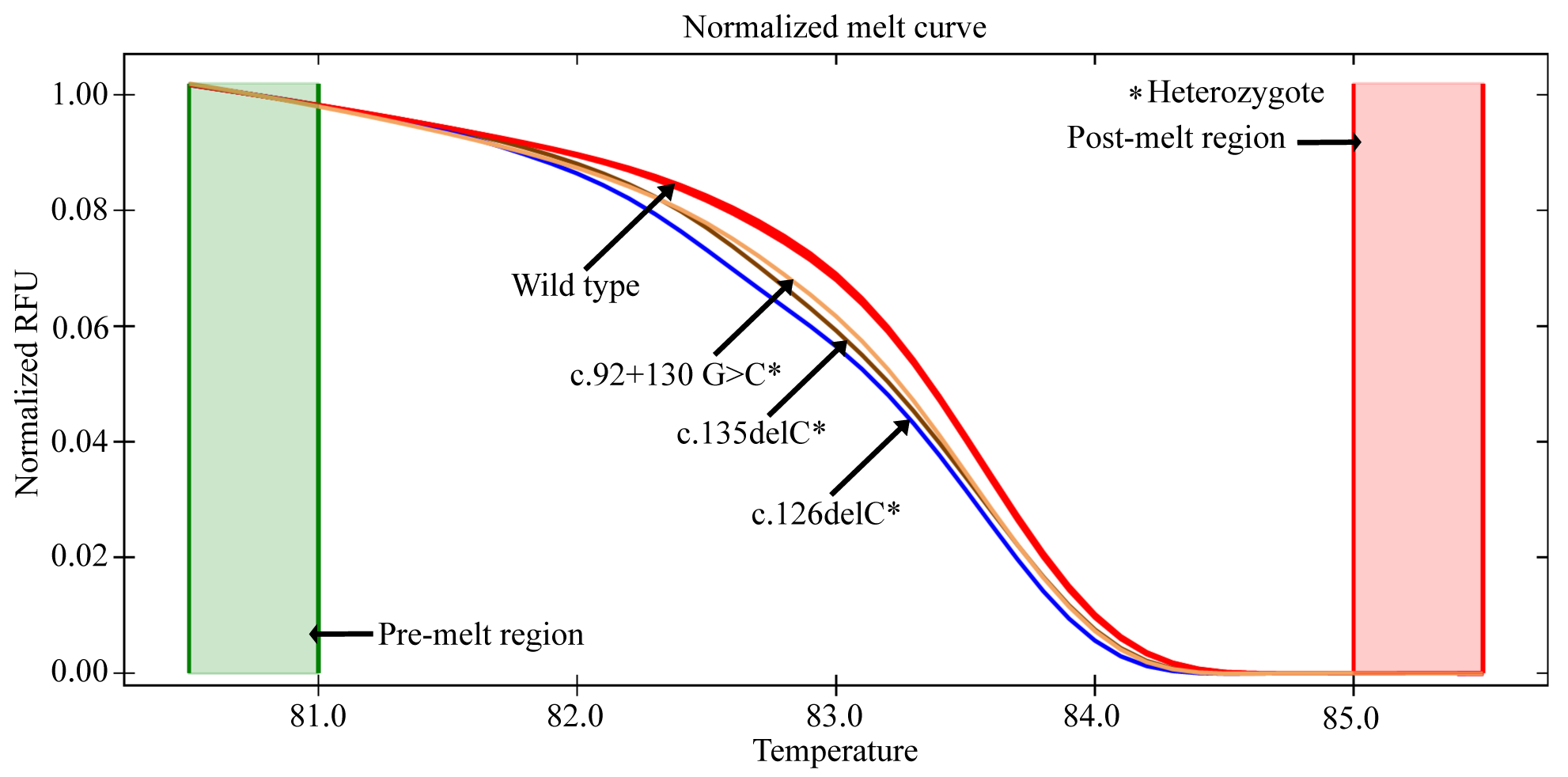

Supplement: Supplementary file 5 — Normalized melt curve patterns of heterozygous c.92 + 130G > C, c.126delC and c.135delC mutations. (TIFF 189 kb) [file 12863_2017_594_MOESM5_ESM.tif]

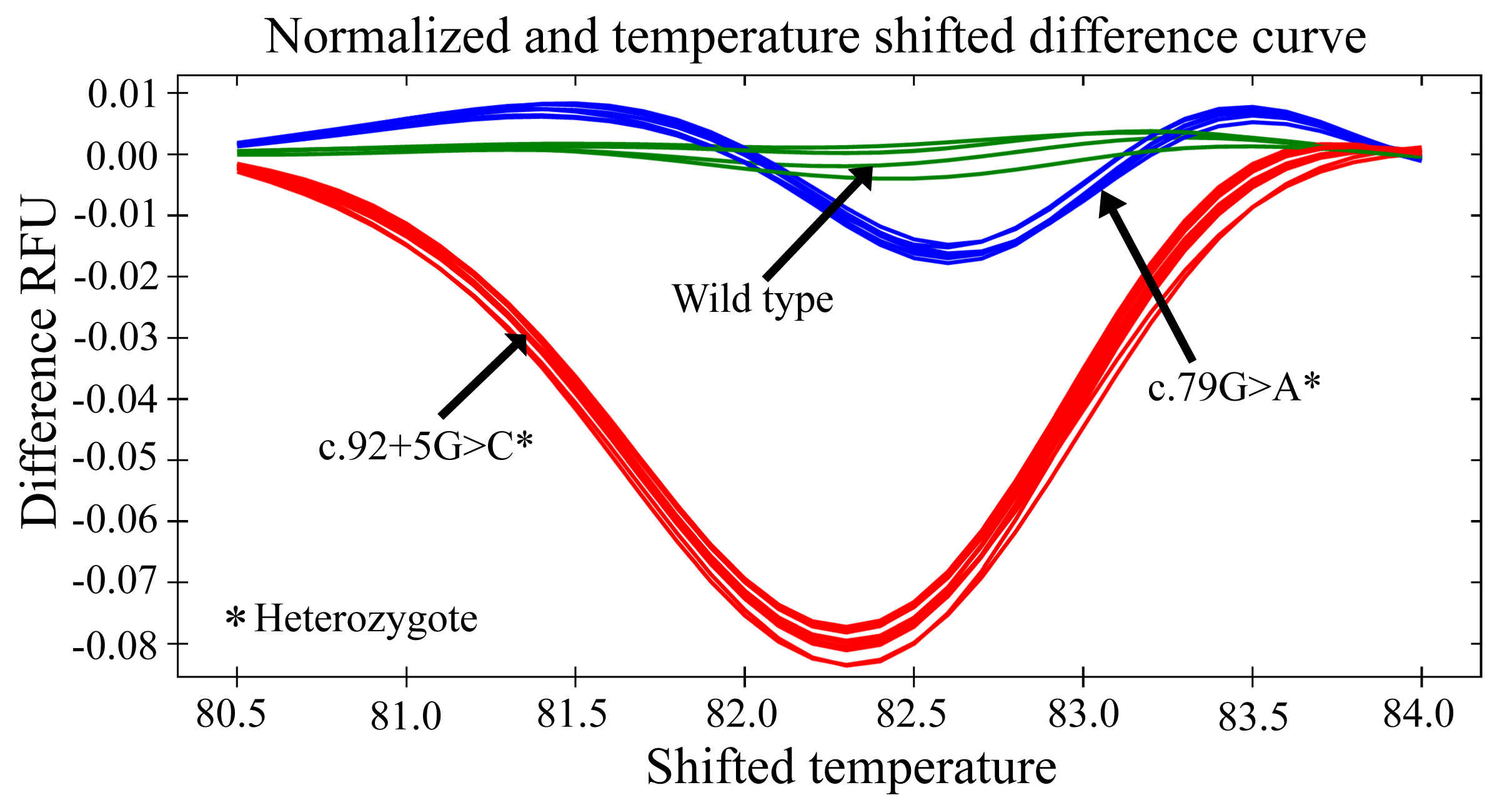

Supplement: Supplementary file 6 — Temperature shifted difference curves of unknown carrier parents subjected to HRM analysis by 1st set of primers. (TIFF 530 kb) [file 12863_2017_594_MOESM6_ESM.tif]

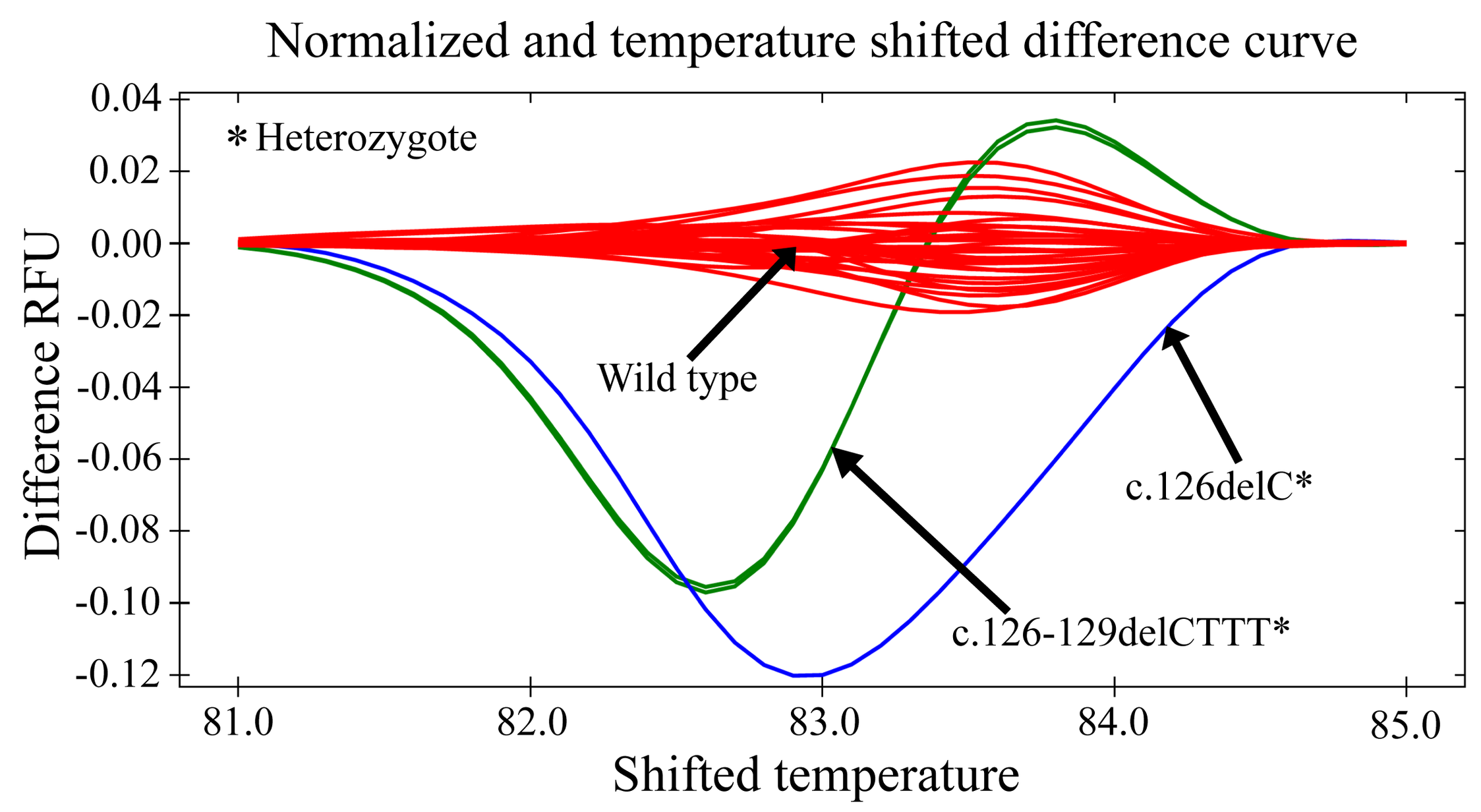

Supplement: Supplementary file 7 — Temperature shifted difference curves of unknown carrier parents subjected to HRM analysis by 2nd set of primers. (TIFF 487 kb) [file 12863_2017_594_MOESM7_ESM.tif]
